# Benefits of soil biochar amendments to tomato growth under saline water irrigation

**DOI:** 10.1038/s41598-018-33040-7

**Published:** 2018-10-03

**Authors:** Dongli She, Xiaoqin Sun, Agbna H. D. Gamareldawla, Elshaikh A. Nazar, Wei Hu, Khaembah Edith, Shuang’en Yu

**Affiliations:** 1 Key Laboratory of Efficient Irrigation-Drainage and Agricultural Soil-Water Environment in Southern China, College of Agricultural Engineering, Hohai University, Nanjing, 210098 China; 2 State Key Laboratory of Soil Erosion and Dryland Farming on the Loess Plateau, Research Center of Soil and Water Conservation and Ecological Environment, Chinese Academy of Sciences & Ministry of Education, Yangling, 712100 China; 3 New Zealand Institute for Plant & Food Research Limited, Private Bag 4708, 8140 Christchurch, New Zealand

## Abstract

Biochar amendments have been used in agriculture to improve soil fertility and enhance crop productivity. A greenhouse experiment was conducted to test the hypothesis that biochar amendment could also enhance the productivity of salt-affected soils. The trial was conducted over two consecutive growing seasons to investigate the effect of biochar amendment (four application rates as: B_1_ = 0%, B_2_ = 2%, B_3_ = 4%, and B_4_ = 8% by mass of soil) on yield and quality of tomatoes grown in a silt loam soil using non-saline water (I_0_ = 0.7 dS m^−1^) and saline water (I_1_ = 1 dS m^−1^; I_2_ = 3 dS m^−1^) irrigation. Furthermore, the study investigated the mechanism by which biochar addresses the salt stress on plant. The results showed that soil productivity as indicated by the vegetative growth and tomato yield components was adversely and significantly affected by saline water irrigation (*P* < 0.05). Tomato yield decreased from 689 ± 35.6 to 533 ± 79.0 g per plant as salinity of irrigation water increased from I_0_ to I_2_. Then, biochar amendment increased vegetative growth, yield, and quality parameters under saline irrigation water regimes, and ameliorated the salt stresses on crop growth. The highest (8.73 ± 0.15 and 4.10 ± 0.82 g kg^−1^) and the lowest (8.33 ± 0.08 and 2.42 ± 0.76 g kg^−1^) values of soil pH and soil organic matter were measured at B_4_I_0_ and B_1_I_2_ treatments, respectively. Also, the highest rate of biochar amendment combining with non-saline water irrigation (B_4_I_0_) produced tomato with the highest plant photosynthetic (17.08 ± 0.19 μmol m^−2^ s^−1^) and transpiration rate (8.16 ± 0.18 mmol H_2_O m^−2^ s^−1^). Mechanically, biochar amendment reduced transient sodium ions by adsorption and released mineral nutrients such as potassium, calcium, and magnesium into the soil solution. Therefore, biochar amendments have the potential in ameliorating salt stress and enhancing tomato production.

## Introduction

Water shortage represents a serious risk to global food security^1–3^. The total global water withdrawal for agricultural, domestic and industrial use is expected to increase by 23% from 1995 to 2025^4^. Irrigated agricultural production in a number of the foremost populated areas of the world, such as China and Pakistan are expected to face extreme water crisis in the near future.

Irrigation is hailed as an intervention to enable agricultural production in areas where rainfall is inadequate, but scarcity of fresh water means some farmers are forced to irrigate plants using poor quality water^5^. This is particularly so where saline water is irrigated and then exacerbating the problem on agricultural fields that suffer the effects of salinization. Currently, about 33% of the global irrigated lands are affected by salinization^6^. Salinity affects plant growth by influencing plant physiological processes including photosynthesis and transpiration^7^. Salt stress decreased soil water potential (osmotic stress), causing an ion toxicity and consequently plant death. Decreased osmotic stress reduces water uptake by plants, which usually through closing leaf stomata and decreasing transpiration, and this negatively affects plant growth by decreasing plant photosynthesis^8^. Therefore, a direct correlation of plant growth with soluble salt concentration and duration of stress has been identified^9^.

The impact of salt stress and approaches to diminish the negative effects has been a subject of a number of studies. Techniques involved in these studies range from management options to identifying plant characteristics that help mitigate the effects of salinity. For example, Jouyban^10^ investigated the use of different techniques i.e. scraping, flushing, and leaching to remove excess salt from root zone of plants. Others have targeted on the utilization of various irrigation techniques in decreasing salinity^11^. Improvement of salt tolerance/resistance is another technique used to address the constraint of soil salinity^12^. Researchers have employed methods to improve plant salt tolerance, including inoculating seeds with halotolerant plant-growth-promoting rhizobacteria, using plant growth regulators and developing salt resistant cultivars^13–15^. However, these approaches can be unprofitable, limited by the high costs and labor-consuming requirements of that are involved in order to counter the salinization problems. More recently, applications of organic conditioners have become a more sustainable and popular approach for enhancing crop productivity in salt-affected soils^5^.

Recent work has elaborated the potentiality of biochar to enhance soil fertility and improve crop productivity^16^. Many studies conducted in different parts of the world have reported improved soil water holding capacity, nutrient availability to plants, and plant productivity as a result of using biochar amendment^1,5,17–19^. Schmidt^20^ reported that a fourfold increase in pumpkin production after soil amended with biochar when mixed with and without cow urine. Kamman^21^ gave a molecular interpretation about the positive effect of biochar on crop production, where he found that development of acid and basic functional groups and organo-mineral complexes on the biochar-matrix surfaces which responsible for nutrients retention. Joseph^22^ also reported that organic coating on biochar explained its ability on nutrients retention. However, a few researches had been done to evaluate/investigate the potential of biochar amendment (BA) in reducing the soil soluble salt under saline water (SW) irrigation as well as enhancing fruit quality. For example, Usman^5^ used conocarpus biochar under SW irrigation to investigate the changes of soil nutrient availability and tomato growth. Additionally, Lashari^23^ concluded that BA increased the plant growth, biomass, and yield and also increased photosynthesis, nutrient uptake, although under salt stress. The feedstock of biochar is of the main factor determining the effects of BA on plant growth. Therefore, the aims of this study were to investigate 1) the effects of wheat straw biochar soil amendment under SW irrigation on growth as well as physiology, yield, fruit quality of tomato; 2) the capability of BA to alleviate salt stress.

## Results

### Effects of BA and SW irrigation on selected soil properties

The analysis of variance results indicated that both BA and SWirrigation significantly (*P* < 0.05) influenced soil EC, SOM and pH as presented in Table 1. The pattern across treatments is greater EC with greater biochar composition, irrespective of irrigation treatment. For example, in 2014, the EC values for the B_1_ treatment increased in the order 0.20 ± 0.07, 0.85 ± 0.07, and 1.64 ± 0.15 dS m^−1^ for the I_0_, I_1_, and I_2_ irrigation treatments, respectively, while the corresponding values for the B_4_ treatment were 0.82 ± 0.07, 1.40 ± 0.21, and 2.25 ± 0.07 dS m^−1^. Similarly, soil pH and SOM increased significantly (*P* < 0.05) by increasing BA rate. For instance, in 2014 and 2015, SOM for I_0_B_1_ was 2.7 ± 0.08 and 2.71 ± 0.07 g kg^−1^, and these increased to, respectively 3.67 ± 0.69 and 4.10 ± 0.82 g kg^−1^ in I_0_B_4_ when the soil amended with 8% biochar.

**Table 1:** Mean values of soil electrical conductivity (EC), pH and soil organic matter (SOM) content for different biochar amendments and irrigation regimes during the 2014 and 2015 tomato growing seasons.

Irrigation regime	Biochar treatment	EC (dS m^−1^)		pH		SOM (g kg^−1^)	
		2014	2015	2014	2015	2014	2015
I_0_	B_1_	0.20^cD^	0.57^cC^	8.44^aB^	8.46^aB^	2.70^aC^	2.71^aD^
	B_2_	0.45^cC^	0.75^cB^	8.65^aA^	8.69^aA^	3.30^aB^	3.40^aC^
	B_3_	0.72^cB^	0.72^cB^	8.68^aA^	8.71^aA^	3.51^aA^	3.81^aB^
	B_4_	0.82^cA^	0.94^cA^	8.70^aA^	8.73^aA^	3.67^aA^	4.10^aA^
I_1_	B_1_	0.85^bD^	1.05^bD^	8.41^bB^	8.42^bB^	2.50^bC^	2.63^bC^
	B_2_	1.18^bC^	1.61^bC^	8.54^bA^	8.55^bA^	2.80^bC^	3.10^bB^
	B_3_	1.28^bB^	1.90^bB^	8.56^bA^	8.56^bA^	3.14^bB^	3.34^bB^
	B_4_	1.40^bA^	2.10^bA^	8.56^bA^	8.58^bA^	3.44^bA^	3.71^bA^
I_2_	B_1_	1.64^aD^	1.90^aC^	8.31^cA^	8.33^cB^	2.30^cC^	2.42^cC^
	B_2_	1.82^aC^	1.99^aC^	8.40^cA^	8.42^cA^	2.40^cB^	2.70^cB^
	B_3_	2.15^aB^	2.40^aB^	8.43^cA^	8.44^cA^	2.45^cB^	2.73^cB^
	B_4_	2.25^aA^	2.72^aA^	8.45^cA^	8.46^cA^	2.76^cA^	2.90^cA^
Biochar		***	***	ns	ns	***	***
Salinity		***	***	*	*	**	**
Interaction		ns	ns	ns	ns	ns	ns

Note: In each column, different uppercase and lowercase letters indicate a significant difference among the biochar application rates and irrigation salinity levels, respectively, at *P* < 0.05 (Least significant difference, LSD); Analysis of variance (ANOVA): ns, not significant; *, **, and ***, denote significance at *P* ≤ 0.05, *P* ≤ 0.01, and *P* ≤ 0.001, respectively. B_1_, B_2_, B_3_ and B_4_ represent mixtures of soil with 0%, 2%, 4% and 8% of biochar by mass. I_0_, I_1_ and I_2_ represent irrigation water salinity of tap water, 1 and 3 dS m^−1^.

**Figure 1:**
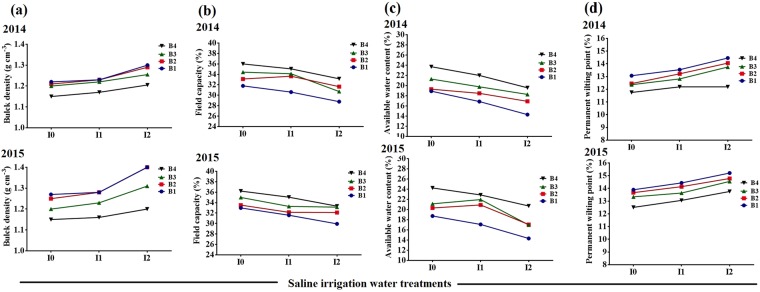
Bulk density, Bd (**a**); Field capacity, FC (**b**); Available water content, AWC (**c**) and Permanent wilting point, PWP (**d**) for different irrigation water salinity and biochar treatments for season 2014 and 2015. B_1_, B_2_, B_3_ and B_4_ represent mixtures of soil with 0%, 2%, 4% and 8% of biochar by mass. I_0_, I_1_ and I_2_ represent irrigation water salinity of tap water, 1 and 3 dS m^−1^.

Bulk density (B_d_) increased significantly in SW irrigation treatments compared to I_0_, and decreased with increasing BA rate (Fig. 1). Treatment I_2_ resulted in 13% and 14% lower B_d_ under the B_4_ biochar compared to the respective non-biochar control, for 2014 and 2015 seasons, respectively. The soil field capacity (FC) was also affected significantly by both SW irrigation and BA treatments. It was decreased significantly in SW treatment compared to I_0_ treatment. However, FC was greater in all BA treatments compared to non-biochar. Figure 1 also shows the soil permanent wilting point (PWP) as influenced by SW irrigation and BA treatments. Soil PWP increased significantly by SW irrigation. However, BA decreased PWP in both I_1_ and I_2_ soil compared to their non-biochar control. Available water content (AWC) for plant decreased significantly under I_1_ and I_2_ compared to I_0_, and increased with the increased rate of BA, for all the SW treatments (Fig. 1). Maximum AWC was observed in the combination of B_4_I_0_.

### The effects of BA and SW irrigation on plant growth

The measured plant growth parameters were significantly (*P* < 0.05) affected by both BA and SW irrigation as shown in Table 2. These growth parameters generally increased significantly with increases in the BA rate and decreased significantly with increases salinity of SW (*P* < 0.05). This was reflected by greater biomass observed for I_0_B_4_ treatments in both growing seasons. However, among all the measured growth parameters, there were no significant interaction effects of BA and SW.

**Table 2:** Mean values of various plant growth parameters for different saline water irrigation regimes and biochar amendments during the 2014 and 2015 tomato growing seasons.

Irrigation regime	Biochar treatment	FAGB (g)		DAGB (g)		FBGB (g)		DBGB (g)		LRWC (%)	
		2014	2015	2014	2015	2014	2015	2014	2015	2014	2015
I_0_	B_1_	240.2^aD^	302.9^aD^	127.5^aD^	190.5^aD^	25.40^aC^		6.32^aB^		92.72^aD^	94.00^aC^
	B_2_	264.3^aC^	327.3^aC^	146.6^aC^	153.8^aC^	26.45^aC^	ND	6.33^aB^	ND	93.80^aC^	95.08^aB^
	B_3_	289.7^aB^	352.7^aB^	156.7^aB^	219.7^aB^	27.88^aB^		7.22^aA^		94.12^aB^	95.07^aB^
	B_4_	307.3^aA^	383.6^aA^	165.7^aA^	228.7^aA^	29.75^aA^		7.58^aA^		95.48^aA^	96.10^aA^
I_1_	B_1_	181.6^bD^	299.9^bD^	125.2^bD^	188.2^bD^	22.09^bD^		6.18^bC^		91.52^bC^	92.14^bC^
	B_2_	248.0^bC^	331.0^bC^	125.5^bC^	199.5^bC^	25.14^bC^	ND	6.88^bB^	ND	92.04^bB^	92.99^bB^
	B_3_	285.5^bB^	348.5^bB^	133.8^bB^	192.9^bB^	25.11^bC^		6.71^bB^		92.59^bB^	94.54^bB^
	B_4_	300.6^bA^	372.3^bA^	146.1^bA^	209.1^bA^	27.65^bA^		6.92^bA^		94.29^bA^	95.24^bA^
I_2_	B_1_	172.0^cD^	251.5^cD^	57.49^cD^	143.5^cD^	20.90^cD^		5.70^cB^		90.61^bC^	90.90^cC^
	B_2_	181.6^cC^	258.0^cC^	61.53^cC^	147.5^cC^	22.80^cC^		6.14^cB^		91.57^bB^	92.47^cB^
	B_3_	192.3^cB^	287.6^cA^	70.69^cB^	163.7^cB^	24.67^cA^	ND	6.34^cA^	ND	92.02^bB^	92.64^cA^
	B_4_	239.9^cA^	286.9^cB^	67.78^cA^	209.6^cA^	24.20^cA^		6.92^cA^		92.89^bA^	93.51^cA^
Biochar		***	***	***	***	***	ND	*	ND	**	***
Salinity		**	***	**	***	***	ND	**	ND	**	***
Interaction		ns	ns	ns	ns	ns	ND	ns	ND	ns	ns

Note: fresh above- ground biomass (FAGB), dry above- ground biomass (DAGB), fresh below- ground biomass (FBGB), dry below- ground biomass (DBGB), and leaf relative water content (LRWC). In each column, different uppercase and lowercase letters indicate a significant difference among the biochar application rates and irrigation salinity levels, respectively, at P < 0.05 (Least significant difference, LSD); Analysis of variance (ANOVA): ns, not significant; *, **, and ***, denote significance at P ≤ 0.05, P ≤ 0.01, and P ≤ 0.001, respectively. ND, no data. B_1_, B_2_, B_3_ and B_4_ represent mixtures of soil with 0%, 2%, 4% and 8% of biochar by mass. I_0_, I_1_ and I_2_ represent irrigation water salinity of tap water, 1 and 3 dS m^−1^.

**Figure 2:**
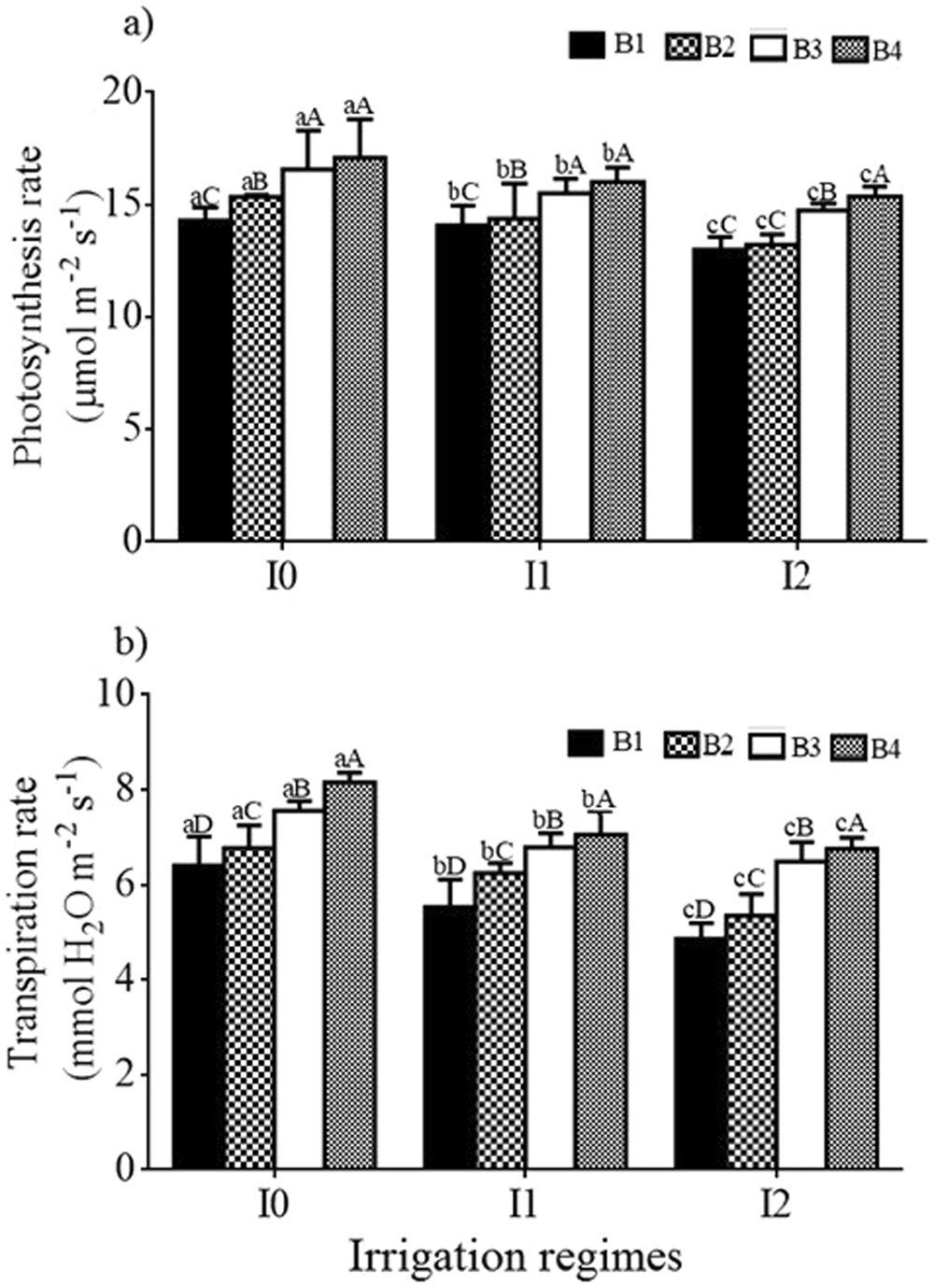
Rates of (**a**) photosynthesis and (**b**) transpiration for different irrigation water salinity and biochar treatments for season 2014. Different uppercase and lowercase letters indicate a significant difference between different biochar application rates (for a given irrigation regime) and different salinity levels, respectively, at *P* < 0.05. B_1_, B_2_, B_3_ and B_4_ represent mixtures of soil with 0%, 2%, 4% and 8% of biochar by mass. I_0_, I_1_ and I_2_ represent irrigation water salinity of tap water, 1 and 3 dS m^−1^.

The Pn and Tr rates were influenced by BA rates and SW irrigation (Fig. 2). Plants irrigated by saline water had significantly lower leaf photosynthetic compared with the control. Within each irrigation treatment, addition of biochar resulted in significant increases in photosynthetic rate. The highest (17.08 ± 0.19 μmol m^−2^ s^−1^) and lowest (13.0 ± 0.25 μmol m^−2^ s^−1^) Pn rates were observed for treatments I_0_B_4_ and I_2_B_1_, respectively. Transpiration rate followed a similar trend with I_0_B_4_ recording the highest value (8.16 ± 0.18 mmol H_2_O m^−2^ s^−1^) while the lowest one (4.85 ± 0.27 mmol H_2_O m^−2^ s^−1^) was recorded under I_2_B_1_. The interaction effects of BA and SW on the Pn and Tr rates were statistically not significant.

### Effects of BA and SW irrigation on tomato yield

**Figure 3:**
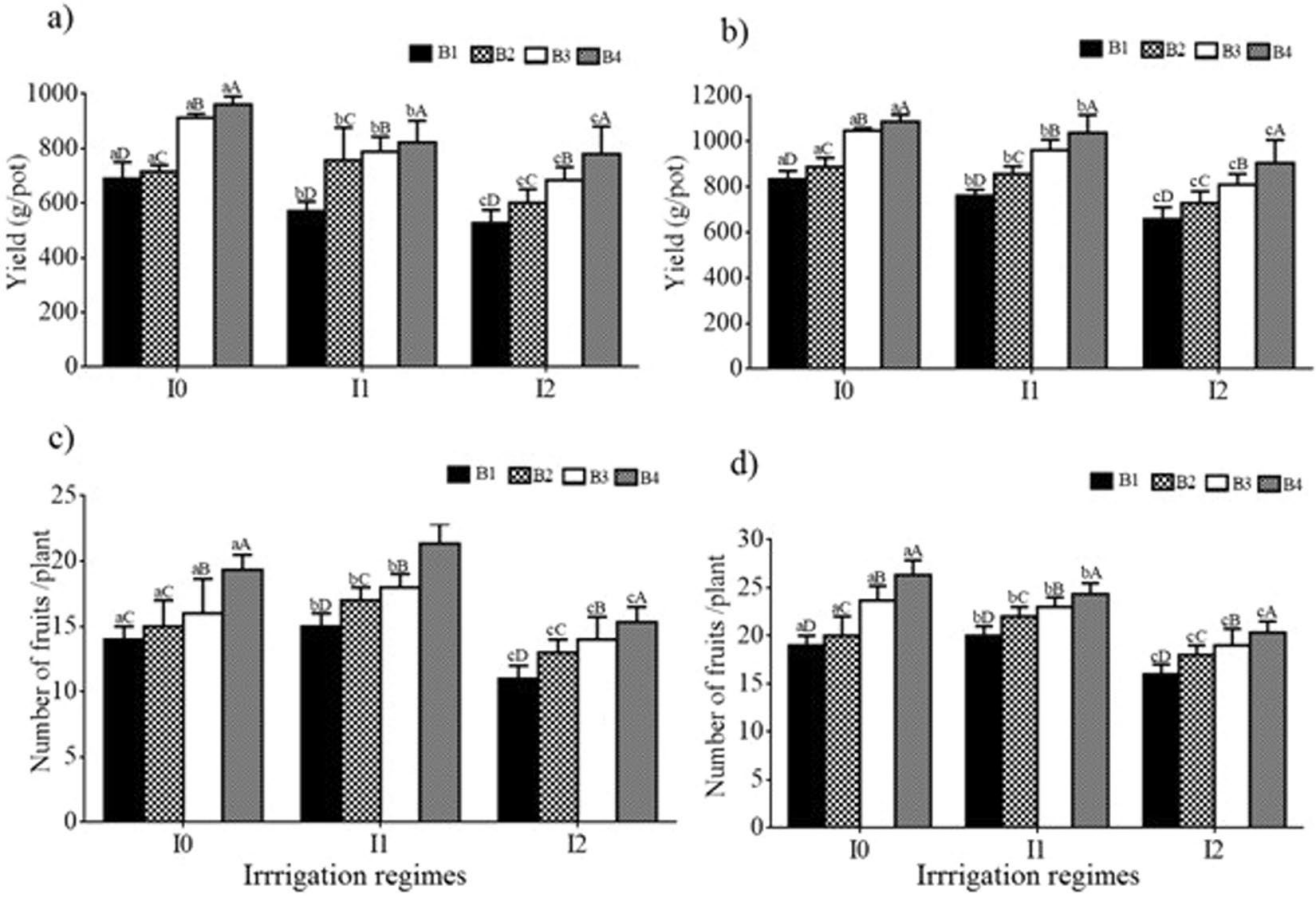
Yield (**a**,**b**) and number of fruits per plant (**c**,**d**) in 2014 and 2015, respectively, for different irrigation water salinity and biochar treatments. For each year, different uppercase and lowercase letters indicate a significant difference among different biochar application rates (for a given irrigation regime) and salinity levels, respectively, at P < 0.05. B_1_, B_2_, B_3_ and B_4_ represent mixtures of soil with 0%, 2%, 4% and 8% of biochar by mass. I_0_, I_1_ and I_2_ represent irrigation water salinity of tap water, 1 and 3 dS m^−1^.

Both BA rate and SW irrigation significantly (*P* < 0.05) influenced tomato yield and the number of fruits per plant (NFP). In both growing seasons, these two yield parameters significantly increased with decreases in irrigation water salinity and increased with increases in BA rate (Fig. 3). For example, in the 2014 control irrigation treatment (I_0_), the B_4_ treatment resulted in an increase of 6 more fruits plant^−1^ and 272 g greater mass than the B_1_ treatment. Increased salinity had a negative effect on yield. For the B_1_ treatment in 2014 for example, the yield decreased from 689 ± 35.6 to 533 ± 79.0 g plant^−1^ as salinity increased from I_0_ to I_2_, which was due to the decreased NFP from 21 to 15. There was no interaction significant (*P* > 0.05) effect of BA and SW on yield.

### Fruit quality as affected by BA and SW irrigation

Generally, the concentrations of total soluble solid (TSS), soluble sugar content (SS), titratable acidity (TA), vitamin C content (VC), and fruit color index (CI) increased with either BA rate increasing or SW irrigation (Table 3). Overall, there were no significant (*P* > 0.05) interaction effects of BA and SW irrigation on fruit quality parameters. Significant differences in the TSS content were observed among both BA and SW irrigation treatments. The results elaborated that the I_2_B_4_ combination gave the greatest values of TSS and VC while I_0_B_1_ had the lowest values for these quality measures. Statistically there were no significant effects on CI value, but numerically increases in SW decreased the CI value while increases in the BA rate got the opposite results. In all cases, the SS content of fruits significantly (*P* < 0.05) increased with increases in SW and BA in both seasons as displayed in Table 3. Significant (*P* < 0.05) differences in the TA content were realized among both the BA and SW irrigation treatments, where highest and lowest TA contents values were found in the treatments I_2_B_4_ and I_0_B_1_, respectively.

**Table 3:** Mean values of tomato quality parameters including total soluble solids (TSS), soluble sugar (SS), titratable acids (TA), vitamin C (VC), color index (CI), and sugar/acid content ratio (SS/TA) for different saline water irrigation treatments and biochar amendments during the 2014 and 2015 growing seasons.

Irrigation regime	Biochar treatment	TSS (Brix %)		VC (mg 100 g^−1^)		TA (g 100 g^−1^)		SS (g 100 g^−1^)		SS/TA		CI	
		2014	2015	2014	2015	2014	2015	2014	2015	2014	2015	2014	2015
I_0_	B_1_	4.0^cC^	5.0^cC^	4.5^cC^	4.7^cD^	0.35^cC^	0.33^cC^	1.14^cC^	1.12^cD^	3.3^aA^	3.5^aA^	1.9^aA^	1.96^aA^
	B_2_	5.0^cB^	5.3^cB^	4.7^cC^	5.0^cC^	0.36^cB^	0.37^cB^	1.16^cB^	1.18^cC^	3.2^aB^	3.2^aB^	1.91^aA^	1.97^aA^
	B_3_	5.5^cA^	5.5^cA^	4.9^cB^	5.2^cB^	0.39^cA^	0.40^cA^	1.20^cA^	1.20^cB^	3.0^aC^	3.0^aC^	1.93^aA^	1.97^aA^
	B_4_	5.5^cA^	5.7^cA^	5.3^cA^	5.5^cA^	0.40^cA^	0.41^cA^	1.20^cA^	1.24^cA^	3.0^aC^	3.0^aC^	1.95^aA^	1.99^aA^
I_1_	B_1_	5.5^bC^	6.0^bA^	4.9^bC^	5.3^bD^	0.37^bD^	0.39^bD^	1.19^bC^	1.28^bD^	3.2^cA^	3.3^cA^	1.86^bA^	1.94^bA^
	B_2_	5.8^bB^	6.0^bA^	5.1^bB^	5.5^bC^	0.41^bC^	0.45^bC^	1.24^bB^	1.29^bC^	3.0^cB^	2.9^cA^	1.88^bA^	1.95^bA^
	B_3_	6.2^bA^	6.2^bA^	5.2^bB^	5.7^bB^	0.44^bB^	0.47^bB^	1.31^bA^	1.31^bB^	3.0^cB^	2.8^cB^	1.89^bA^	1.97^bA^
	B_4_	6.3^bA^	6.0^bA^	5.6^bA^	5.9^bA^	0.46^bA^	0.481^bA^	1.31^bA^	1.34^bA^	2.9^cC^	2.8^cB^	1.90^bA^	1.98^bA^
I_2_	B_1_	6.7^aB^	6.7^aB^	5.7^aC^	6.1^aC^	0.41^aC^	0.42^aC^	1.33^aC^	1.38^aC^	3.2^bA^	3.3^bA^	1.85^cA^	1.90^cA^
	B_2_	6.7^aB^	6.8^aB^	6.1^aB^	6.2^aC^	0.45^aB^	0.46^aB^	1.38^aB^	1.42^aB^	3.1^bB^	3.1^bB^	1.85^cA^	1.90^cA^
	B_3_	7.0^aA^	7.0^aA^	6.4^aB^	6.7^aB^	0.50^aA^	0.51^aA^	1.39^aB^	1.42^aB^	2.8^bC^	2.8^bC^	1.86^cA^	1.91^cA^
	B_4_	7.3^aA^	7.0^aA^	6.8^aA^	6.9^aA^	0.50^aA^	0.52^aA^	1.43^aA^	1.46^aA^	3.1^bB^	2.8^cB^	1.88^cA^	1.93^cA^
Biochar		*	ns	**	**	***	***	*	*	*	***	ns	ns
Salinity		***	***	***	***	***	***	***	***	*	*	ns	ns
Interaction		ns	ns	ns	ns	ns	ns	ns	ns	ns	*	ns	ns

Note: In each column, different uppercase and lowercase letters indicate a significant difference among the biochar application rates and irrigation salinity levels, respectively, at *P* < 0.05 (Least significant difference, LSD); Analysis of variance (ANOVA): ns, not significant; *, **, and ***, denote significance at *P* ≤ 0.05, *P* ≤ 0.01, and *P* ≤ 0.001, respectively. B_1_, B_2_, B_3_ and B_4_ represent mixtures of soil with 0%, 2%, 4% and 8% of biochar by mass. I_0_, I_1_ and I_2_ represent irrigation water salinity of tap water, 1 and 3 dS m^−1^.

## Discussion

### Effects on soil properties

Results of this study clearly indicated that soil chemical properties (i.e., EC and SOM) are affected by BA and SW irrigation. The significant increase in soil EC with higher rates of BA is consistent with previous findings^24^, and is due to soluble salts in the biochar entering to the soil solution (Table 4)^24–26^. The SOM decreased with the SW irrigation for a given rate of BA. This indicated that high salinity probably decreased the biochar decomposition rate, which would also reduce the rate at which nutrients were released^27^. These impacts of increased salinity, both due to the BA and the SW irrigation, would typically be unfavorable to plant productivity^28^. However, increasing BA significantly increased SOM which is essential for water retention and nutrients in the soil for plants, thus, alleviating the negative effects of salinity.

**Table 4:** Mean values of soil cation contents (g kg^−1^) for different biochar application rates (B) and irrigation water salinity levels (I).

Irrigation regime	Biochar treatment	Ca	K	Mg	Na
I_0_	B_1_	0.07^dC^	0.01^bC^	0.01^cB^	0.05^eA^
	B_2_	0.35^dB^	0.09^cB^	0.04^bA^	0.04^dA^
	B_3_	0.49^eA^	0.23^dA^	0.05^cA^	0.02^fA^
I_1_	B_1_	0.17^cC^	0.02^bC^	0.02^bB^	0.67^dA^
	B_2_	0.37^cB^	0.10^cB^	0.04^bA^	0.23^cB^
	B_3_	0.51^eA^	0.25^dA^	0.06^cA^	0.14^eC^
I_2_	B_1_	0.19^cC^	0.03^bC^	0.02^bC^	1.23^cA^
	B_2_	0.39^bB^	0.12^bB^	0.04^bB^	0.53^cB^
	B_3_	0.58^dA^	0.30^cA^	0.07^bA^	0.14^eC^
Biochar		***	**	**	***
Salinity		*	ns	*	***
Interaction		**	ns	*	**

Note: In each column, different uppercase and lowercase letters indicate a significant difference among the biochar application rates and irrigation salinity levels, respectively, at *P* < 0.05 (Least significant difference, LSD); Analysis of variance (ANOVA): ns, not significant; *, **, and ***, denote significance at *P* ≤ 0.05, *P* ≤ 0.01, and *P* ≤ 0.001, respectively. B_1_, B_2_, and B_3_ represent mixtures of soil with 0%, 2%, and 4% of biochar by mass. I_0_, I_1_ and I_2_ represent irrigation water salinity of tap water, 1 and 3 dS m^−1^.

The positive effects of BA on soil physical properties were well documented. The results indicated a significant decrease in soil B_d_, and an increase in SWC, FC, PWP, and AWC in the biochar-amended soils, even at the low biochar application rate, under both SW and non-SW irrigation treatments (Fig. 1). Moreover, salt stress adversely affected soil productivity, as indicated by the higher B_d_ and PWP, and lower FC and AWC of the soil under SW irrigation; however, this suppressing effects on soil physical properties tended to decline with BA, especially at the high biochar application rates^29^. Soil water retention capacity (AWC and FC) is very important property with respect to plant growth^30^. Laird^29^ described that the biochar amended soil retained 15% more moisture contents as compared controlled treatment, which was consistent with our results.

### Effects on plant growth

Negative impacts of increased salt stress on plant growth were reflected by the responses of measured plant parameters. Both Pn and Tr rates declined due to salt stress, and this was linked to changes in water potential of leaf in previous studies. As described by Kazuhiro^31^, leaf water potential controls stomatal conductance which affects Tr and Pn, and affects water uptake by plant driven by the potential difference between leaf and soil water^19,32^. Salt stress caused stomata closure which reduce the CO_2_/O_2_ ratio in leaves and inhabit CO_2_ fixation^33^. The net effect of BA to soil irrigated with SW on these processes was favorable in this study, since plant Tr rates were significantly higher when tomato grown under I_2_B_4_ combination comparing to I_0_B_1_. This indicated that BA can be utilized to compensate for the negative effects of salinity on leaf Tr rates, especially at sufficiently high biochar application rate (Fig. 2).

Previous studies illustrated that organic amendments are capable to enhance soil characteristics and plant growth^34^. This typically, was due to the improvement of soil environment, such as by enhancing macro-nutrient and water availability^35,36^, which also could increase the plant resistance to salt stresses^36–38^. This study elaborated that all of the growth parameters were adversely impacted by SW irrigation. This occurs because salts negatively impact plants by both inducing physical drought by osmotic effects that impede water transport in the plants and by ion toxicity^39^. In contrast, BA significantly increased plant growth and the yield, which implied that biochar, could ameliorate the adverse effects of salt stress on plants. The improved vegetative growth in soils treated with biochar was inline with the findings of Hossain^40^, who found that BA improved the vegetative growth of cherry tomato.

### Effects on fruit quality

Although increasing the salinity in irrigation water decreased tomato yield, it increased significantly fruit quality. In other studies on tomato, similar trends were observed whereby TSS, vitamin C, and acidity increased with increases EC in the soil solution^41–44^. Increased salinity of irrigation water might cause the plant to regulate metabolic processes that decreased the production of sucrose and organic acids and/or reallocated them to different plant parts, particularly to the fruits, and thus increased the concentration gradient of sucrose from leaves to fruits^45^.

Similarly, values of the fruit quality parameters increased significantly as BA rate increased. Of particle note is that BA increased the TA content in all the irrigation treatments. These enhancements in fruit quality may be attributed to the BA which affected the root distribution^46^. Although rooting depth was not measured in this study, the BA increased root biomass. Usman^5^ obtained similar results, who found that BA increased both TSS and VC slightly under both SW and non-SW irrigation treatments. Moreover, Akhtar^44^ and Agebna^16^ reported that BA improved the quality of tomato under deficit irrigation.

### Mechanisms by which BA ameliorates salt stress on plants

This study provided evidences that BA can be utilized in salt-affected soils and/or when irrigation water is of low quality. It is probable that BA ameliorated negative impacts of salinity in the tomato plants by three main mechanisms: 1) reducing transient sodium ions by adsorption; 2) releasing mineral nutrients such as potassium, calcium, and magnesium into the soil solution (Table 4); and 3) decreasing osmotic stress by improving the soil AWC^18^. Novak^47^ stated that BA has strong absorptive characteristics binding refer to its high porosity, surface area and cation exchange capacity. By adsorbing toxic ions, especially sodium, and/or by releasing more beneficial ions^18^, BA can therefore reduce the negative effects of salt stress on plants, either by decreasing the exposure of plants to stress agents or by mitigating the stress responses of plants. Soil water content can be increased because that BA increased soil water holding capacity, especially increasing the proportion of larger pores where water is held at lower potentials allowing plants to uptake water more readily (Fig. 1)^47^. Increasing water availability might also interpret the alleviation of salt stress observed^47^. Some studies on photosynthetic responses to biochar additions have illustrated the increased water use efficiency at the leaf or whole plant scale^36–38^.

## Conclusions

Saline water irrigation resulted remarkable decrement in tomato growth parameters and physiological processes such as Pn and Tr rates. Soil BA reduced the adverse impacts of salt stress, particularly at higher biochar application rates. The positive effects of BA on tomato growth were due to its capability to adsorb sodium ions and thus reducing its hazards and releasing mineral nutrients such as potassium, calcium, and magnesium into soil solution. Therefore, BA could be used on salt affected soils to improve tomato yield and quality, which would apply to saline-sodic soils elsewhere in the world. In particular, BA has the potential to be widely used combination with SW irrigation in agricultural production to struggle with fresh water crisis.

## Materials and Methods

### Study site

The experiment was carried out in a greenhouse from March 2014 to August 2015 at the Water-Saving Park of Hohai University (31°57′N, 118°50′E), China. The climate in the area is humid subtropical monsoon with annual mean pan evaporation and precipitation of 900 and 1073 mm, respectively. The mean daily temperature inside the greenhouse was 27.8 °C. More details about this area can be found in Agebna^16^. The physio-chemical soil properties are displayed in Table 1.

### Experimental design

To evaluate the combination effects of soil BA and SW irrigation on tomato growth, the experiment was conducted as a 3 × 4 factorial laid out in a completely randomized design with three replicates. Irrigation (I) water with three different salt concentrations were evaluated; I_0_ (control; tap water had electrical conductivity (EC) of 0.7 dS m^−1^), I_1_ (EC = 1 dS m^−1^) and I_2_ (EC = 3 dS m^−1^). Sodium chloride (NaCl) was mixed with tap water to prepare the irrigation water with a given salinity. Soil BA treatments comprising four different proportions of biochar in biochar-mixtures i.e. 0%, 2%, 4%, and 8% and designated as B_1_, B_2_, B_3_, and B_4_, respectively, were evaluated. The wheat straw biochar was used in this study, which was pyrolyzed at 350–550 °C. Typically, 30% dry matter of the wheat straw would be converted into biochar^48^. The initial biochar properties are presented in Table 5.

**Table 5:** Basic physical and chemical properties of biochar and the soil in the study area.

Soil Properties	Soil	Biochar
pH	7.7	9.9
Electrical Conductivity (dS m^−1^)	1.42	1.0
Ca (g kg^−1^)	0.16	0.016
Mg (g kg^−1^)	0.07	1.3
Cl (g kg^−1^)	0.11	1.44
HCO_3_ (g kg^−1^)	0.20	0.85
total N (g kg^−1^)	1.8	59
total P (g kg^−1^)	0.66	144.3
total K (g kg^−1^)	0.4	115
CEC (cmol kg^−1^)	14.94	217
Bulk density (g cm^−3^)	1.35	0.40
Field capacity (%)	25.8	—
Silt (%)	30.1	—
Sand (%)	50.2	—
Clay (%)	19.7	—

### Agronomic practices

Tomato (*Lycopersiconesculentum* Mill, Yazhoufenwang), which is a pink tomato, infinite growth variety, was chosen as the experimental crop. Seeds were sown on March 6 (2014, season 1) and March 10 (2015, season 2) in a nursery, respectively. The Seedlings were transplanted when plants had reached four-leaf stage (April 14 in 2014 and April 19 in 2015, respectively). During the growing period, the weigh method was utilized to verify water losses and then irrigating replace the lost amount of water. The total amounts of water irrigated in each treatment were 147.2 mm and 142.3 mm for 2014 and 2015 growing seasons, respectively. The agricultural applications during the tomato growth periods were the same as Agebna^16^.

### Measurements of soil physio-chemical properties

At the end of each growing seasons (August 12 in 2014 and August 5 in 2015, respectively), soil physio-chemical properties were measured. For each treatment, a disturbed soil sample (∼1 kg) was collected from the upper 20 cm layer, and air-dried. The air-dried samples were packed down to pass through a 1-mm mesh before being extracted using a 5:1 soil: leaching liquor to analyze soil pH, EC and cation contents (K^+^, Na^+^, Ca^2+^, Mg^2+^)^49–54^. Cation exchange capacity was determined using the ammonium acetate method^52^. Soil organic matter (SOM) content was measured using oil bath K_2_Cr_2_O_7_ titration method^49,52^. Soil total nitrogen (TN) was determined using the method described by Bremneran^53^, and the total phosphorus (TP) was determined following Olsen and Sommers^54^. An undisturbed soil core was removed from the surface (0–20 cm) layer at each treatment for soil bulk density (B_d_) measurement^55^. The core sampler had an internal diameter of 5 cm and height of 5 cm^56^. Field capacity (FC) and soil permanent wilting point (PWP) were determined as described by Michael^57^. Soil available water content (AWC) was obtained by subtracting values of PWP from FC^58^.

### Measurements of tomato growth and yield

Tomato growth parameters, including fresh above- (FAGB) and below- (FBGB) ground biomass, dry above- (DAGB) and below- (DBGB), were measured at the end of each growing seasons, following the details at Agebna^16^. Plant leaf relative water content (LRWC) was measured at one day closing to the middle of the growing season (June 13 in 2014 and July 18 in 2015), utilizing the method describe by Smart^59^. Two mature leaves in plant upper canopy of each treatment were selected to measure plant photosynthesis (Pn) and transpiration (Tr) rates. Four measurements were carried out in the growing season of 2014 (May 23, June 11, July 6 and July 17, respectively), using a portable photosynthesis system (Li-Cor, Lincoln, NE, USA) during the period of 09:00–12:00 h. Mean Pn and Tr rates were calculated from the four measurements.

Tomato fruits were harvested from July 18 to August 12 in 2014 and July 14 to August 5 in 2015. The number of fruits/plant (NFP) was accounted and weighed immediately to determine fruit mass (Yield). Fruits were homogenized using a blender, and chemical composition was quantified to determine fruit quality parameters, including parameters of total soluble solid (TSS, Brix %), titratable acidity (TA, g 100 g^−1^), vitamin C content (VC, mg 100 g^−1^ fresh mass, as ascorbic acid), soluble sugar content (SS, g 100 g^−1^) and fruit color index (CI)^60–64^.

### Statistical analysis

All the treatments were subjected to a two way analysis of variance (ANOVA) using the MSTATC statistical package^65,66^. The Least Significant Difference (LSD; *P* ≤ 0.05) was used to determine differences between means.
